# Supplementary material for: Improving Interpretation Consistency of Serum Capillary Electrophoresis by Development of Quantitative Graphic Indexes
Source: Int J Mol Sci. 2024 Nov 14;25(22):12240. doi: 10.3390/ijms252212240 (PMC11594780; doi:10.3390/ijms252212240)
Supplement: Supplementary file 1 [file ijms-25-12240-s001.zip › Table S1.pdf]

Table S1. Separate reference intervals of the proposed capillary zone electrophoresis-immunosubtraction indexes by age and sex

	Males less than 65 years old	Females less than 65 years old	Males with 65 years old or more	Females with 65 years old or more
Sharpness index				
γ zone	(-5, -1)	(-4, -1)	(-5, -1)	(-5, -1)
β2 zone	(-17, -3)	(-15, -3)	(-16, -3)	(-16, -4)
Light chain index				
γ zone	(1.09, 2.79)	(1.14, 2.7)	(1.06, 2.78)	(0.96, 2.75)
β2 zone	(0.53, 2)	(0.5, 1.84)	(0.32, 2.13)	(0.4, 2)
Immunoglobin G index				
γ zone	(31, 414)	(51, 365)	(39, 450)	(34, 430)
β2 zone	(-7, 45)	(-5, 39)	(-8, 59)	(-9, 50)
Immunoglobin A index				
γ zone	(-8, 36)	(-10, 31)	(-9, 40)	(-11, 37)
β2 zone	(2, 100)	(4, 81)	(0, 113)	(0, 110)
Immunoglobin M index				
γ zone	(-19, 43)	(-16, 41)	(-19, 48)	(-17, 46)
β2 zone	(-15, 30)	(-12, 27)	(-15, 33)	(-16, 32)

[*] All reference intervals are expressed with lower and higher reference limits in parentheses.
