# Supplementary figures and images for: Improving Interpretation Consistency of Serum Capillary Electrophoresis by Development of Quantitative Graphic Indexes
Source: Int J Mol Sci. 2024 Nov 14;25(22):12240. doi: 10.3390/ijms252212240 (PMC11594780; doi:10.3390/ijms252212240)

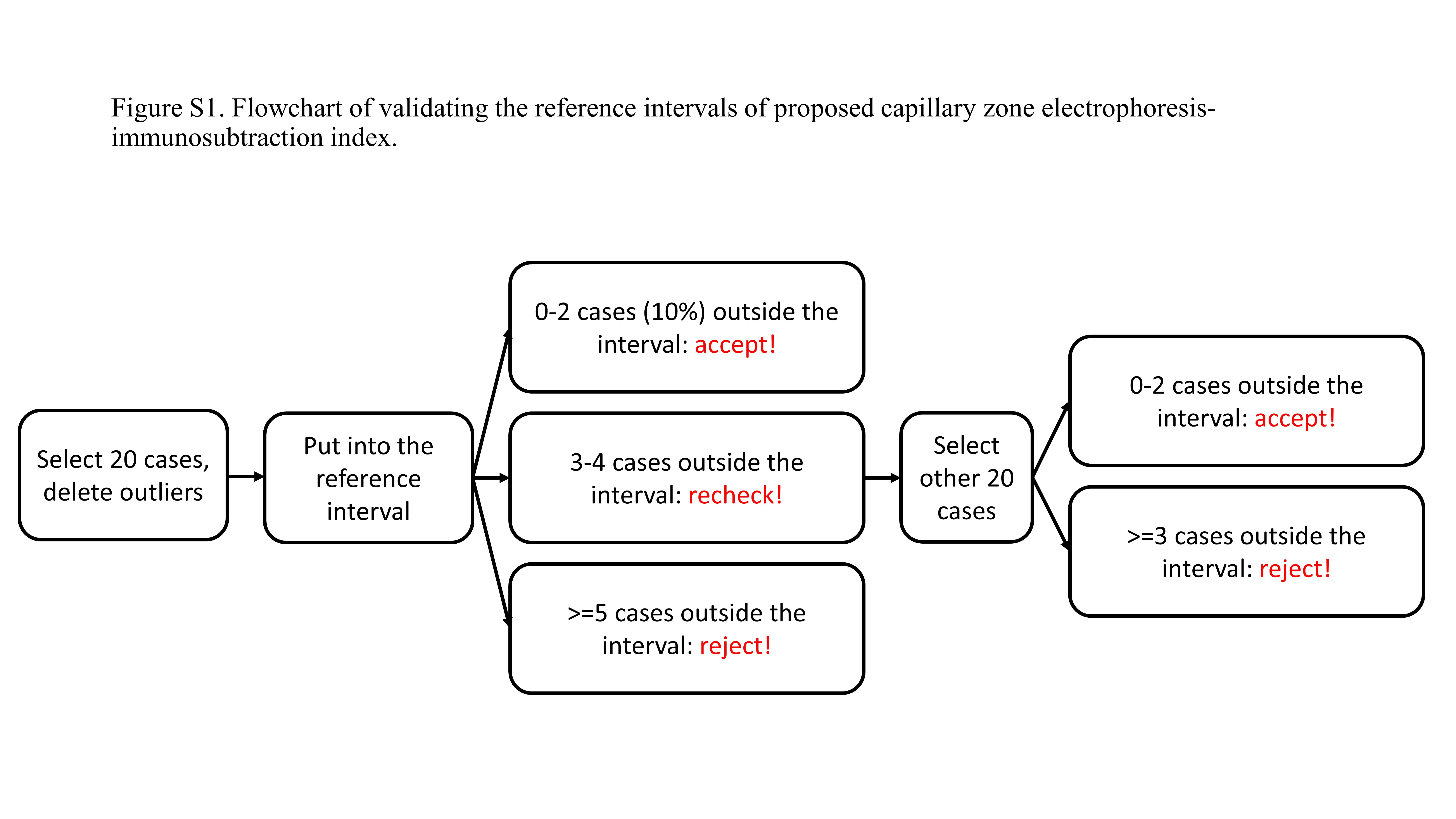

Supplement: Supplementary file 1 [file ijms-25-12240-s001.zip › Figure S1.jpg]
